# Potential Role of Transferrin and Vascular Cell Adhesion Molecule 1 in Differential Diagnosis Among Patients with Tauopathic Atypical Parkinsonian Syndromes

**DOI:** 10.3390/diagnostics15212676

**Published:** 2025-10-23

**Authors:** Natalia Madetko-Alster, Dagmara Otto-Ślusarczyk, Marta Struga, Patryk Chunowski, Piotr Alster

**Affiliations:** 1 Department of Neurology, Medical University of Warsaw, Kondratowicza 8, 03-242 Warsaw, Poland; patryk.chunowski@wum.edu.pl (P.C.); piotr.alster@wum.edu.pl (P.A.); 2 Department of Biochemistry, Medical University of Warsaw, Banacha 1, 02-097 Warsaw, Poland; dagmara.otto@wum.edu.pl (D.O.-Ś.); marta.struga@wum.edu.pl (M.S.)

**Keywords:** atypical Parkinsonian syndrome, progressive supranuclear palsy, corticobasal syndrome, inflammation, transferrin, VCAM-1

## Abstract

**Background/Objectives:** Transferrin is a multi-task protein commonly known for binding iron; however, it is involved in multiple crucial processes, including antimicrobial activity, the growth of different cell types, differentiation, chemotaxis, the cell cycle, and cytoprotection. Vascular cell adhesion molecule 1 (VCAM-1) is a cell surface glycoprotein which participates in inflammation and the trans-endothelial movement of leukocytes. Neither transferrin nor VCAM-1 has been studied in the context of progressive supranuclear palsy (PSP) or corticobasal syndrome (CBS). This study aimed to evaluate the utility of transferrin and VCAM-1 assessment for the in vivo examination of tauopathic atypical Parkinsonian syndromes. **Methods**: This study included 10 patients with clinically probable PSP-RS, 10 with clinically probable PSP-P, and 8 with probable CBS. Patients’ blood and urine were collected and analyzed. Twenty-four serum samples (from twelve males and twelve females) were obtained from age-matched healthy volunteers. Peripheral blood inflammatory ratios, including the neutrophil-to-lymphocyte ratio, the platelet-to-lymphocyte ratio, the neutrophil-to-monocyte ratio, the neutrophil-to-high-density lipoprotein ratio, and the monocyte-to-high-density lipoprotein ratio, were calculated. VCAM-1 and transferrin concentrations were measured in the serum and urine. The urinary biomarker results are not included in the main analysis due to the absence of a control group. **Results**: The highest concentrations of transferrin in the serum were observed in patients with PSP-P, followed by PSP-RS and CBS. Statistically significant differences were found between PSP-P and healthy controls (*p* < 0.0001) and PSP-RS and healthy controls (*p* < 0.0001). The highest levels of serum VCAM-1 were observed in the PSP-P group. Significant differences were found between PSP-P and healthy controls (*p* < 0.0001), PSP-P and CBS (*p* < 0.001), and PSP-RS and healthy controls (*p* < 0.001). Serum VCAM-1 levels were negatively correlated with the NLR in CBS patients (*p* < 0.03; *r* = −0.74). Serum transferrin levels were negatively correlated with the NHR in CBS patients (*p* < 0.04; *r* = −0.64). ROC curve analyses were conducted to evaluate the diagnostic utility of serum transferrin and VCAM-1 in distinguishing tauopathic APS patients from controls. Transferrin showed excellent diagnostic performance, with an AUC of 0.975 (95% CI: 0.888–0.999; *p* < 0.0001), a sensitivity of 96.4%, and a specificity of 95.8% at the optimal cut-off (>503.0). VCAM-1 demonstrated good accuracy, with an AUC of 0.839 (95% CI: 0.711–0.926; *p* < 0.0001), a sensitivity of 75.0%, and a specificity of 91.7% at the optimal cut-off (>463.9). **Conclusions:** The obtained results indicate the potential role of transferrin and VCAM-1 in the pathogenesis of tauopathic APSs and highlight the need for further exploration in this field.

## 1. Introduction

Atypical Parkinsonian syndromes (APSs) neuropathologically defined as tauopathies include progressive supranuclear palsy (PSP) and corticobasal degeneration (CBD). According to the current diagnostic criteria for PSP and CBD [1,2], a definite diagnosis requires postmortem neuropathological verification; therefore, clinical diagnoses are probable at most. Corticobasal syndrome (CBS) includes multiple underlying pathologies aside from CBD, such as PSP, Alzheimer’s disease, frontotemporal dementia (FTD), globular glial tauopathy, Lewy body disease (LBD), and Creutzfeldt–Jakob disease (CJD) [3]; however, most of those pathologies remain connected with tau.

APSs currently remain incurable, with a poor response to symptomatic treatment. Despite several decades of research, the exact mechanism leading to neurodegeneration remains unclear with multiple hypotheses having been proposed, including environmental factors such as age, diet, lifestyle, exposure to toxins, metabolic impairment, oxidative stress, genetically determined susceptibility, or vascular coupling [4]. Among all theories concerning the mechanisms of neurodegeneration, the neuroinflammatory hypothesis seems to be the most justified and universal. The first suggestions considering the coexistence of inflammatory processes and neurodegeneration were published in the 1980s by McGeer [5]. This neuropathological study described the presence of reactive microglia in the brains of patients with Parkinson’s disease (PD), Parkinsonism with dementia, and Alzheimer-type dementia [5]. More recent papers have confirmed microglial activation among patients with Alzheimer’s disease (AD) and PSP [6], infiltration of immunocompetent cells in the brain with multiple system atrophy (MSA) [7], or the presence of proinflammatory cytokines in the serum of patients with PS and other APSs [8,9].

Peripheral inflammation in neurodegenerative Parkinsonian syndromes is generalized enough to be reflected in non-specific blood morphology parameters such as the monocyte-to-high-density lipoprotein ratio (MHR) [10], the neutrophil-to-lymphocyte ratio (NLR) [11], and the platelet-to-lymphocyte ratio (PLR) [12], among others.

Some data indicate the existence of significant differences between concentrations of interleukin 1β (IL1β) and interleukin 6 (IL6) in serum and cerebrospinal fluid (CSF) among patients with PSP–Richardson’s syndrome (PSP-RS) and PSP–Parkinsonism predominant (PSP-P), with the lowest concentrations of interleukins observed among the PSP-RS group [13]. This could indicate the presence of distinct neuroinflammatory patterns or a neuroprotective role of increased inflammatory activity, which could cause the differences between PSPS phenotypes [13]. The exact impact of neuroinflammation in the context of the neurodegenerative process remains ambiguous. Some data indicate its negative impact and role in promoting neurodegeneration [14], while imaging studies suggest the existence of at least a partially beneficial impact of inflammation on the intensity of atrophy in specific regions of the brain [15].

Transferrin is a multi-task protein commonly known for binding iron. It is highly polymorphic with more than 30 different species [16], some of which have been reported to increase the risk of developing a neurodegenerative disease, e.g., the C2 allele of transferrin was confirmed to increase the risk of AD among carriers of the C282Y allele of the haemochromatosis gene, especially among apolipoprotein E4 carriers [17]. The underlying mechanism could be related to oxidative stress caused by an excess of redox-active iron. However, this correlation was not confirmed among the Korean population [18]. Transferrin is involved in multiple crucial processes, including iron binding and transportation, antimicrobial activity, different cell type growth, differentiation, and chemotaxis, as well as cell cycle and cytoprotection [16].

Vascular cell adhesion molecule 1 (VCAM-1) is a cell surface glycoprotein participating in inflammation and the trans-endothelial movement of leukocytes [19]. Expression of VCAM-1 can be found in multiple cell types—e.g., the endothelium, dendritic cells, bone marrow stromal cells, macrophages, and astrocytes—and can be induced by proinflammatory cytokines, reactive oxygen species, or stress [19]. The interaction between VCAM-1 and integrin on leukocytes’ surface disrupts endothelial cell junctions (due to actin remodeling), enabling leukocytes to infiltrate injured tissue [20]. VCAM-1 is continuously shed from the cell surface by membrane metalloproteinase ADAM17, which causes the presence of plasma soluble VCAM1 [21]. VCAM-1 participates in autoimmune disorders; it has been linked to cardiovascular diseases, stroke, cancer, and psychiatric disorders such as schizophrenia and bipolar disorder [20,22,23].

The role of vascular abnormalities and iron metabolism deviations in the pathophysiology of atypical Parkinsonisms [3,24], though previously indicated, has not been sufficiently analyzed. In this context, the assessment of VCAM1 and transferrin, though based on non-specific parameters, may provide a perspective on tendencies that are likely feasible in further investigations. To the best of our knowledge, neither transferrin nor VCAM-1 has been previously studied in the context of PSP or CBS.

## 2. Materials and Methods

Twenty-eight patients were included in this study, fifteen males and thirteen females, aged from 55 to 80 years, with a clinical diagnosis of tauopathic APS. The diagnoses were made based on contemporary criteria of diagnosis of PSP and CBS [1,2]. Additionally, the authors evaluated subtypes of PSP, i.e., PSP-RS and PSP-P. Ten patients with clinically probable PSP-RS, ten with clinically probable PSP-P, and eight with probable CBS were included in this study. All diagnoses were made during hospitalization in the Department of Neurology, the Medical University of Warsaw, by an experienced movement disorder specialist. All study participants were Non-Hispanic Caucasians. The disease duration varied from 3 to 6 years in each case. In terms of clinical severity, MDS-UPDRS part III scores varied from 18 to 30 points, average 22 points; none of study participants received dopaminergic therapy at the time of evaluation and at least 6 weeks prior to the examination. None of the study participants received any medications that could have influenced the analyzed data. Apart from amlodipine (5 mg), low-dose aspirin (75 mg daily), ramipril (5 mg), and sertraline (25 mg), no other medications were prescribed, therefore this information was not included in the analysis.

The exclusion criteria included previous vascular diseases of the central nervous system (CNS), previous head injuries, congenital cognitive deficits, a history of a neoplastic disease, active or recent (8 weeks prior to inclusion) infection, a history of an autoimmune disorder, the presence of nutritional deficiencies, or organ failure.

Every study participant provided written consent and underwent blood and urine sampling. All samples were collected in the morning, on an empty stomach. Every patient underwent basic laboratory tests, including peripheral blood morphology, levels of C-reactive protein (CRP), procalcitonin, creatinine, electrolytes, and liver parameters. Patients with significant abnormalities suggesting neoplasms, infectious diseases, or organ failure were excluded from this study.

Twenty-four serum samples (twelve males and twelve females) were obtained from age-matched healthy volunteers forming the control group. All samples were evaluated in the Department of Biochemistry of the Medical University of Warsaw. The peripheral inflammatory ratios were calculated based on blood morphology and lipid profiles:

- NLR—neutrophil-to-lymphocyte ratio;
- PLR—platelet-to-lymphocyte ratio;
- NMR—neutrophil-to-monocyte ratio;
- NHR—neutrophil-to-high-density lipoprotein ratio;
- MHR—monocyte-to-high-density lipoprotein ratio.

All ratios were calculated manually by dividing its first component by the second.

### 2.1. Patients’ Blood and Urine Collections

The blood samples (5 mL) were drawn into test tubes without an anticoagulant and then centrifuged. The resulting serum samples were subsequently frozen at −80 °C until analysis. The fresh urine “clear midstream” samples of 20–50 mL were aliquoted and frozen at −80 °C. Repeated freezing and thawing were avoided until analysis was performed. Urine samples did not require any special additives or preservatives.

### 2.2. Vcam-1 and Transferrin Measurements in Patients

The analysis involved determining the levels of vCAM-1 and transferrin using a sandwich solid-phase enzyme-linked immunosorbent assay (ELISA) (Invitrogen, Waltham, MA, USA). The assays were performed according to the manufacturer’s instructions using human vCAM-1 (catalog number KHT0601) and human transferrin (catalog number: EHTF) ELISA kits.

To ensure values fell within the dynamic range of the assay, both serum and urine samples were diluted (typically 1:50) before analysis. The final concentrations were calculated by multiplying the measured value by the dilution factor. Each assay was performed in duplicate. The absorbance readings were measured at 450 nm using a plate reader, and the molecule concentrations were determined from standard curves generated using manufacturer-supplied standards. The standard curves consistently showed excellent linearity (R^2^ > 0.99).

### 2.3. Statistical Analysis of Patient Data

Statistical analysis was performed using GraphPad Prism 9.0 (GraphPad Software, San Diego, CA, USA) and MedCalc Statistical Software version 20.218 (MedCalc Software Ltd., Ostend, Belgium). Variables with a normal distribution are presented as means and standard deviations (SDs). Data normality was assessed using the Shapiro–Wilk test. Correlation analysis between two variables was conducted using Pearson’s correlation. The Mann–Whitney U test was used for comparisons between two groups, while the Kruskal–Wallis test was applied for comparisons involving three or more groups. When the Kruskal–Wallis test was significant, Dunn’s post hoc test was performed. Receiver operating characteristic (ROC) curve analyses were performed to evaluate the diagnostic performance of transferrin and vCAM-1. The area under the curve (AUC) with 95% confidence intervals was calculated, and optimal cut-off values were determined using the Youden index. Statistical significance was defined as a two-tailed *p*-value of less than 0.05.

## 3. Results

### 3.1. Demographic, Hematological, and Biochemical Characteristics

The demographic, clinical, and biochemical characteristics of all groups are summarized in Table 1. No significant differences in mean age were observed between PSP-RS, PSP-P, CBS, and control groups. Hematological parameters (neutrophils, lymphocytes, monocytes, hemoglobin, and platelets) and HDL cholesterol values are provided for all groups. Serum transferrin and VCAM-1 levels are also shown, with notably lower concentrations in CBS patients compared with PSP subgroups and controls.

### 3.2. Inflammatory Ratios

The inflammatory ratios across the study groups are summarized in Table 2. The NLR, PLR, MHR, NMR, and NHR values did not significantly differ between groups. These findings suggest that the systemic inflammatory profiles, assessed using commonly used hematological ratios, were largely comparable across PSP-P, PSP-RS, CBS, and control subjects.

### 3.3. Transferrin Levels in Serum

**Figure 1:**
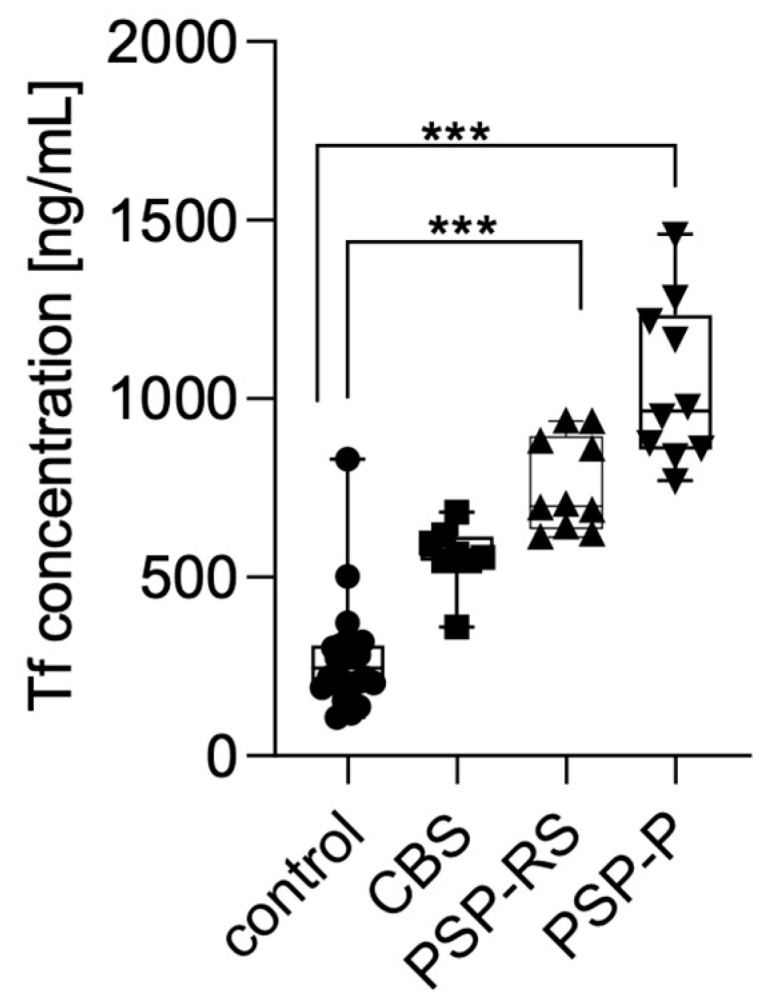
Transferrin levels in the serum among analyzed groups. *** *p* < 0.0001.

Serum transferrin levels were measured using enzyme-linked immunosorbent assay (ELISA) and compared across the analyzed groups. Statistical significance was calculated using analysis of variance followed by Dunne’s post hoc test. The highest concentrations were observed in patients with PSP-P, followed by PSP-RS and CBS. Statistically significant differences were found between PSP-P and healthy controls (*p* < 0.0001) and PSP-RS and healthy controls (*p* < 0.0001). No other statistically significant differences were found. No significant differences were observed between the tauopathic syndromes (CBS, PSP-RS, and PSP-P); moreover, the CBS group did not significantly differ when compared with controls. Results are presented in Figure 1.

### 3.4. VCAM-1 Levels in Serum

**Figure 2:**
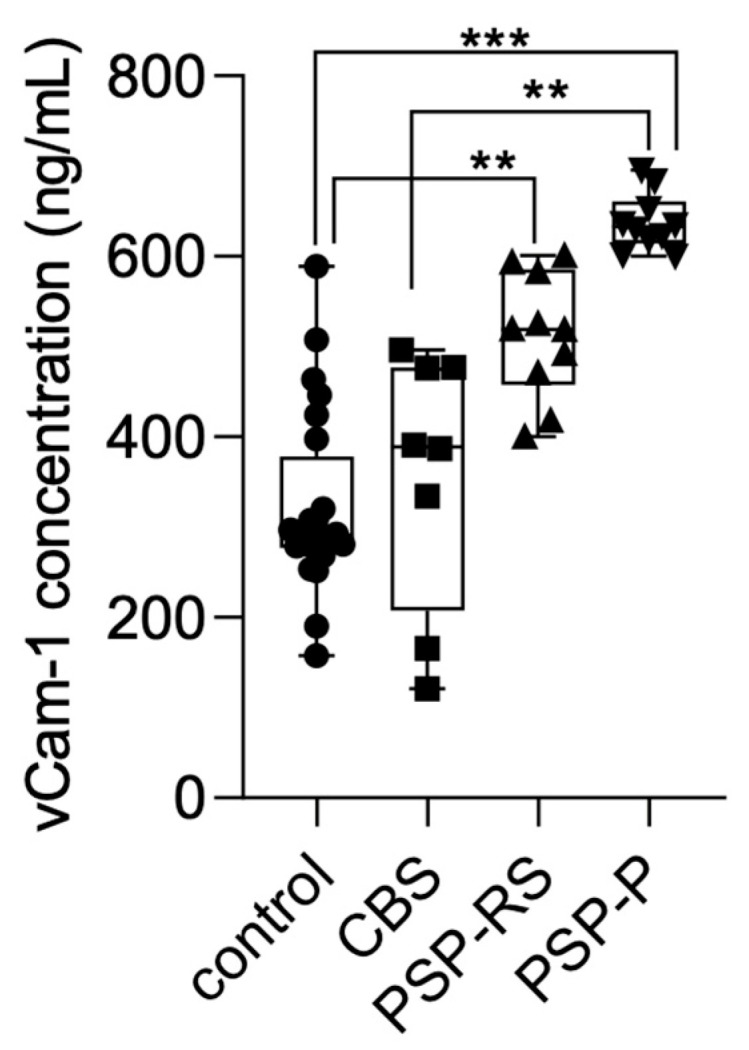
VCAM-1 levels in the serum among analyzed groups. *** *p* < 0.0001 ** *p* < 0.001.

Serum VCAM-1 concentrations were also measured using ELISA and compared across the analyzed groups. Statistical significance was calculated using analysis of variance followed by Dunne’s post hoc test. The highest levels were observed in the PSP-P group. Significant differences were found between PSP-P and healthy controls (*p* < 0.0001), PSP-P and CBS (*p* < 0.001), and PSP-RS and healthy controls (*p* < 0.001). No other statistically significant differences were found. Results are presented in Figure 2.

### 3.5. Correlations with Inflammatory Ratios

This study showed certain significant correlations of the examined factors with non-specific peripheral inflammatory parameters. Statistical analysis of associations between serum biomarker levels (VCAM-1 and transferrin) and peripheral inflammatory ratios revealed the following: serum VCAM-1 levels were negatively correlated with the NLR in CBS patients (*p* < 0.03; *r* = −0.74).

Serum transferrin levels were negatively correlated with the NHR in CBS patients (*p* < 0.04; *r* = −0.64). The correlation was not detected in PSP-P and PSP-RS.

**Figure 3:**
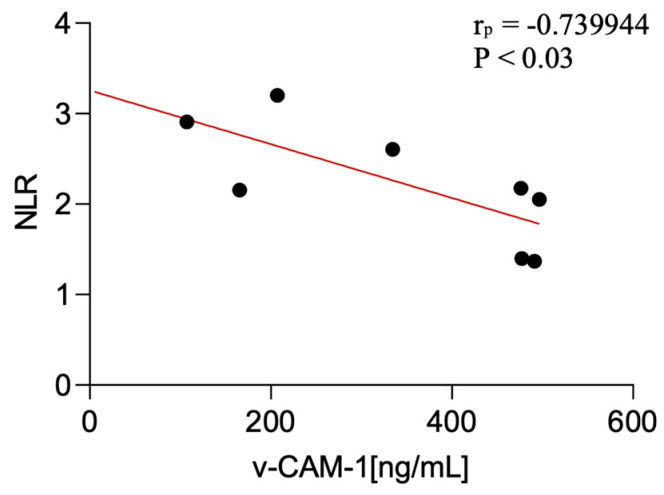
Correlation between serum v-CAM-1 levels and NLR among CBS patients.

**Figure 4:**
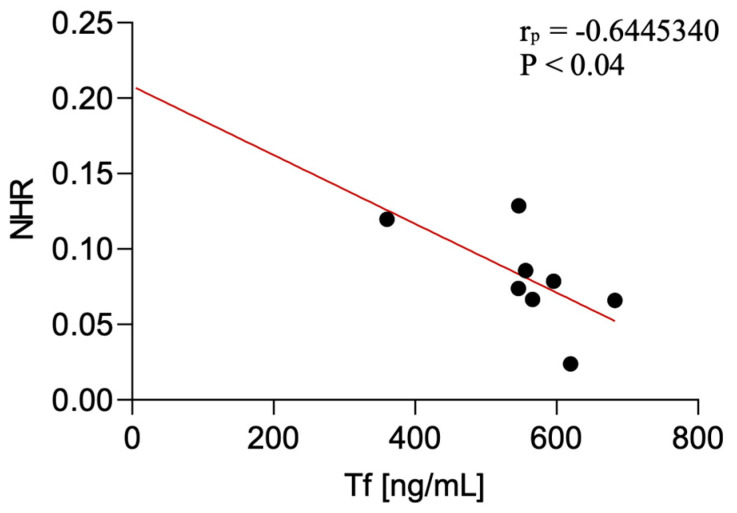
Correlation between serum transferrin levels and NLR among CBS patients.

These findings are presented in Figure 3 (VCAM-1), Figure 4 (transferrin) and Table 3.

The urinary biomarker results have been moved to the Supplementary Material (Supplementary Figures S1, S2, S3A,B and S4A,B, and Tables S1 and S2) and are not included in the main analysis due to the absence of a control group.

All correlation coefficients (*r*) and *p*-values for all analyzed relationships between inflammatory ratios and VCAM-1 or transferrin concentrations in serum and urine are presented in Supplementary Tables S1 and S2 (including significant and non-significant findings for completeness).

### 3.6. Diagnostic Performance (ROC Curve Analysis)

**Figure 5:**
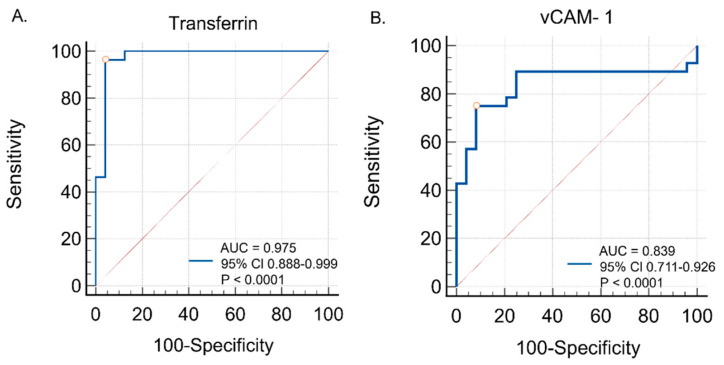
Diagnostic utility of (**A**) serum transferrin and (**B**) serum vCAM-1 in distinguishing tauopathic APS patients from controls—ROC curve analysis.

ROC curve analyses were conducted to evaluate the diagnostic utility of serum transferrin and VCAM-1 in distinguishing tauopathic APS patients from controls. Transferrin showed excellent diagnostic performance, with an AUC of 0.975 (95% CI: 0.888–0.999; *p* < 0.0001), a sensitivity of 96.4%, and a specificity of 95.8% at the optimal cut-off (>503.0). VCAM-1 also demonstrated good accuracy, with an AUC of 0.839 (95% CI: 0.711–0.926; *p* < 0.0001), a sensitivity of 75.0%, and a specificity of 91.7% at the optimal cut-off (>463.9). These findings are illustrated in Figure 5, with detailed data provided in Table 4.

## 4. Discussion

The obtained VCAM-1 and transferrin serum measurements in this preliminary single center study suggest that tauopathic Parkinsonisms are likely to differ when compared with controls; however, their significance in the differentiation of PSP-P, PSP-RS, and CBS seems barely pronounced, as the only significant difference was observed between PSP-P and CBS in serum transferrin. These evaluations concerning CBS should be interpreted cautiously, due to the low number of patients and the likely diverse pathological background.

In vitro studies based on Neuro-2a cells, a mouse neuroblastoma cell line sharing multiple properties with neurons, have indicated that transferrin has a beneficial impact on the cell cycle as it decreases apoptosis and, therefore, promotes neuron survival [25]. Additionally, the study proved the pro-differentiation properties of transferrin. In the context of atypical Parkinsonian syndromes and previously discussed inflammation as a potential factor enhancing neurodegeneration, a study on Neuro-2a cells and microglia co-cultures indicated that transferrin has an impact on microglia, as it caused an increase in the synthesis of anti-inflammatory interleukin 10 (IL-10) and a decrease in the synthesis of proinflammatory tumor necrosis factor (TNFα), interleukin 1 beta (IL-1β), and interleukin 6 (IL-6) [25]. This suggests, as noted by Perez et al. [25], that transferrin modulates microglia toward an M2 phenotype. M2 microglia, sometimes called “alternatively activated,” are associated with the suppression of inflammatory response and tissue repair [26]. In the long-term, dominance of the M1 phenotype has been associated with aging, neurotoxicity, and neurodegeneration [26]; therefore, factors influencing the modification of the microglia phenotype toward M2 could be beneficial. These data may be interpreted as possibly aligning with the results obtained in this study, as the highest concentrations of serum transferrin were found among PSP-P, a phenotype with a relatively mild course. Although transferrin is synthesized by oligodendroglia and choroid plexus [25], the majority of transferrin present in the central nervous system is synthesized in the liver and crosses the blood–brain barrier (BBB) [27]. The authors hypothesize that an increased level of serum transferrin in PSP-P could be a compensatory mechanism, in which increased peripheral transferrin synthesis induces a neuroprotective effect. In this model, the higher concentration of transferrin observed among PSP-RS patients could be explained by compensatory failure or a different mechanism underlying the neurodegenerative process, which is less dependent on microglial activity. The existence of different inflammatory patterns in the course of PSP-P and PSP-RS was previously assessed by the authors of this study in previously published research [13].

The highest concentration of transferrin in urine observed among the CBS population (Supplementary Material) could potentially reflect its multicausal character [3] and the presence of nephrotoxic comorbidities (e.g., diabetes), as it is a marker of subclinical tubular alterations [28].

In Perner et al.’s study concerning PD, plasma VCAM1 levels were significantly higher compared with healthy controls [29]. In PD, VCAM1 levels correlated with Hoehn and Yahr disease stage, part II of MDS-UPDRS, and PDQ-39; however, they did not correlate with age, disease duration, MDS-UPDRS part III, or NMS-Quest [29]. Animal studies indicated that VCAM-1 could play an important role in aging and sustaining neuroinflammation by facilitating leukocyte adhesion and brain endothelial cell inflammation, which results, inter alia, in microglial activation and impaired cognition [30]. A study conducted by the Wyss-Coray group [30] indicated that VCAM1 expression is upregulated during aging or by exposure to aged plasma; however, the exact factors responsible for that effect remain unknown. Interestingly, exposure to young plasma had rejuvenating effects [30]. VCAM-1 involvement in neuroinflammatory sustainment could potentially explain its highest levels among PSP-P and PSP-RS patients compared with healthy controls. The highest concentration of VCAM-1 detected among the PSP-P population aligns with our previously published results concerning IL-1 and IL-6 levels in cerebrospinal fluid (CSF) and serum among the PSP-P population [13]. Relatively low VCAM1 levels among CBS patients (similar to those observed among healthy controls) could partially indicate a different mechanism from that of the inflammatory disease.

The VCAM-1 levels in the analyzed patients could be potentially linked to blood–brain barrier leakiness, which could facilitate neuroinflammation via increased infiltration of leukocytes into the CNS. VCAM-1 levels were associated with microglial activity and p-tau accumulation among patients with chronic traumatic encephalopathy [31]. Serum VCAM-1 was also reported to be increased among patients with schizophrenia [32], which could support the neuroinflammatory hypothesis concerning its pathogenesis [33].

Animal models indicate that misfolded tau protein could influence endothelial features of the BBB, thus causing an increase in the expression of VCAM1, enhancing diapedesis of peripheral immunocompetent cells through the BBB, and promoting inflammation [34].

Animal studies have suggested that interference with VCAM-1 expression intensity—and, therefore, improvement of BBB leakproofness and a reduction in neuroinflammation—could have neuroprotective and anti-aging properties, even with improvement of age-related cognitive decline [35].

Urine levels of vCAM-1 (Supplementary Material) have not been discussed in the context of neurodegeneration. Previous studies suggested its association with nephritis and renal failure; however, the results obtained in this study regarding ratios based on blood evaluation stress its possible links with peripheral inflammation. The positive correlation between urine vCAM-1 and HDL-derived parameters (the NHR and MHR) may arise from studies highlighting the possible significance of HDL in the BBB’s permeability [36]. The possible significance of HDL-derived ratios is also stressed in correlation with urine transferrin; however, in PSP-P, it is a clinical entity with a relatively favorable course. This link, due to the role of transferrin, may partly highlight the significance of iron in the neurodegenerative process and its possible association with inflammation in the pathophysiology of PSP-P [37]. The interpretation of CBS pathophysiology is affected by the fact that it is likely a group of diseases connected by clinical manifestation. In the context of the negative correlations between vCAM-1 and NHR as well as between transferrin and NLR, the fact that the observation can be detected only in CBS—a heterogeneous group likely based on various pathologies—should highlight the necessity of extended analyses rather than excluding the possible significance of inflammation in the pathogenesis [38].

The possible significance of the indicated associations of transferrin and VCAM-1 in this study should be interpreted as a feature encouraging further investigations rather than a conclusive outcome. This pilot study highlights tendencies that possibly contribute to understanding the background of atypical Parkinsonisms—entities affected their by rarity and unrecognized pathophysiology.

### Limitations

This study was limited by the relatively small number of included patients; however, APSs are rare and this study was conducted in only one center. In particular, the very small CBS subgroup (*n* = 8) made the correlation analyses highly sensitive to outliers. These results should, therefore, be interpreted with caution. All diagnoses were made according to clinical criteria without neuropathological verification; however, this was not possible as all study participants remained alive. All data were based on a singular measurement; therefore, this study did not evaluate possible changes in the analyzed factors’ concentrations during disease progression. Another limitation was the lack of urine samples in the control group, which was caused by the outpatient qualification of its members and difficulties in obtaining a sample under these conditions. In this context, the results of the urinary analyses are exploratory and should be interpreted cautiously.

## 5. Conclusions

This study emphasizes the possibly multifactorial (in terms of inflammation) pathogenesis of PSP and CBS. The obtained results were affected by methodological limitations; however, it should be stressed that microglial activation is only one of the multiple inflammatory mechanisms impacting the evolution of these diseases. Although the literature concerning the roles of transferrin and vCAM-1 in the pathogenesis of tauopathic Parkinsonian syndromes is relatively scarce, the results obtained from this study highlight certain tendencies requiring further exploration based on larger groups, including the extended analysis of parameters linked with various inflammatory factors not solely bound to microglial-derived agents. The outcome of this study should be interpreted as an initial point informing further discussions concerning the roles of the evaluated parameters.

## Figures and Tables

**Table 1:** Demographic, clinical and biochemical characteristics of study groups.

Parameter	Control (*n* = 24)	PSP-P (*n* = 10)	PSP-RS (*n* = 10)	CBS (*n* = 8)
Age (years)	66.9 ± 6.2	65.9 ± 9.2	67.3 ± 6.7	69.6 ± 5.4
Neutrophils (×10^9^/L)	5.5 ± 2.1	5.1 ± 1.5	4.1 ± 1.4	3.19 ± 0.67
Lymphocytes (×10^9^/L)	2.1 ± 0.8	1.9 ± 0.8	1.52 ± 0.5	1.59 ± 0.47
Monocytes (×10^9^/L)	0.5 ± 0.2	0.52 ± 0.14	0.44 ± 0.13	0.39 ± 0.10
Platelets (×10^9^/L)	252 ± 73.7	213.1 ± 33.6	213.5 ± 44.7	215.7 ± 56.7
HDL (mg/dL)	40.4 ± 23.1	50.3 ± 12.7	47.1 ± 11.8	44.5 ± 20.3
VCAM-1 serum (ng/mL)	322.7 ± 99	637.8 ± 32.0	472.4 ± 170.8	356.1 ± 142.9
Transferrin serum (ng/mL)	273.1 ± 147	1041.4 ± 227.3	742.8 ± 133.6	559.1 ± 92.7

**Table 2:** Inflammatory ratios across groups (mean and SD).

Ratio	Control (*n* = 24)	PSP-P (*n* = 10)	PSP-RS (*n* = 10)	CBS (*n* = 8)
NLR	2.98 ± 1.32	2.92 ± 1.6	2.86 ± 1.04	2.23 ± 0.65
PLR	143.4 ± 71.2	127.3 ± 59.0	156.4 ± 65.2	155.9 ± 60.5
MHR	0.27 ± 0.13	0.01 ± 0.001	0.01 ± 0.004	0.01 ± 0.006
NMR	11.6 ± 8.5	9.1 ± 2.3	9.1 ± 2.4	8.62 ± 2.1
NHR	0.15 ± 0.09	0.10 ± 0.04	0.09 ± 0.02	0.08 ± 0.01

NLR, neutrophil to lymphocyte ratio, PLR, platelet to lymphocyte ratio, MHR, monocyte to high density lipoprotein ratio, NMR, neutrophile to monocyte ratio, NHR, neutrophil to high density lipoprotein ratio: the data shown are the means  ±  SD.

**Table 3:** Statistically significant correlations of the examined factors with non-specific peripheral inflammatory parameters.

Substance	Ratio	Biological Fluid	APS Subtype	Type of Correlation	Statistical Parameters
vCAM-1	NLR	serum	CBS	negative	*p* < 0.03, *r*_p_ = −0.739944
Transferrin	NHR	serum	CBS	negative	*p* < 0.04, *r*_p_ = −0.6445340

**Table 4:** Diagnostic performance of serum transferrin and vCAM-1 in distinguishing tauopathic APS patients from controls (AUC, 95% CI, *p*-value, optimal cut-off, sensitivity, specificity).

Biomarker	AUC	95% CI	*p*-Value	Optimal Cut-Off	Sensitivity/Specificity
Transferrin	0.975	0.888–0.999	<0.0001	>503.0	96.4%/95.8%
vCAM-1	0.839	0.711–0.926	<0.0001	>463.9	75.0%/91.7%

## Data Availability

The original contributions presented in the study are included in the article. Further inquiries can be directed to the corresponding author.

## Supplementary Materials

The following supporting information can be downloaded at: https://www.mdpi.com/article/10.3390/diagnostics15212676/s1, Figure S1: Transferrin levels in the urine among patients with tauopathic atypical parkinsonian syndrome. * *p* < 0.05, *** *p* < 0.0001; Figure S2: VCAM-1 levels in the urine among patients with tauopathic atypical parkinsonian syndrome. ** *p* < 0.001; Figure S3A: The association between urine vCAM-1 levels and NHR in patients with corticobasal syndrome (CBS), as determined using Pearson’s correlation coefficient (*r*_p_); Figure S3B: The association between urine vCAM-1 levels and MHR in patients with corticobasal syndrome (CBS), as determined using Pearson’s correlation coefficient (*r*_p_); Figure S4A: The association between urine transferrin (Tf) levels and NHR in patients with progressive supranuclear palsy with predominant parkinsonism (PSP-P), as determined using Pearson’s correlation coefficient (*r*_p_); Figure S4B: The association between urine transferrin (Tf) levels and MHR in patients with progressive supranuclear palsy with predominant parkinsonism (PSP-P),as determined using Pearson’s correlation coefficient (*r*_p_); Table S1: Associations between serum vCAM-1 or transferrin levels and peripheral inflammatory ratios; Table S2: Associations between urine vCAM-1 or transferrin levels and peripheral inflammatory ratios.
