# Supplementary material for: Potential Role of Transferrin and Vascular Cell Adhesion Molecule 1 in Differential Diagnosis Among Patients with Tauopathic Atypical Parkinsonian Syndromes
Source: Diagnostics (Basel). 2025 Oct 23;15(21):2676. doi: 10.3390/diagnostics15212676 (PMC12609891; doi:10.3390/diagnostics15212676)
Supplement: Supplementary file 1 [file diagnostics-15-02676-s001.zip › supplementary table S2.pdf]

Table S2. Associations between urine vCAM-1 or transferrin levels and peripheral inflammatory ratios

SUBSTANCE	RATIO	BIOLOGICAL FLUID	APS SUBTYPE	TYPE OF CORRELATION	STATISTICAL PARAMETERS
vCAM-1	NLR	urine	CBS	positive	$p<0.30$, $r_p= 0.417411$
**vCAM-1**	**NHR**	**urine**	**CBS**	**positive**	**$p<0.03$, $r_p= 0.739848$**
vCAM-1	NMR	urine	CBS	positive	$p<0.85$, $r_p= 0.0765805$
vCAM-1	PLR	urine	CBS	positive	$p<0.91$, $r_p= 0.0453929$
**vCAM-1**	**MHR**	**urine**	**CBS**	**positive**	**$p<0.03$, $r_p=0.7452211$**
vCAM-1	NLR	urine	PSP-P	negative	$p<0.45$, $r_p= -0.0785819$
vCAM-1	NHR	urine	PSP-P	positive	$p<0.46$, $r_p= 0.0364559$
vCAM-1	NMR	urine	PSP-P	positive	$p<0.13$, $r_p= 0.379161$
vCAM-1	PLR	urine	PSP-P	negative	$p<0.41$, $r_p= - 0.0785819$
vCAM-1	MHR	urine	PSP-P	negative	$p<0.34$, $r_p= -0.142526$
vCAM-1	NLR	urine	PSP-RS	negative	$p<0.40$, $r_p= -0.297766$
vCAM-1	NHR	urine	PSP-RS	negative	$p<0.45$, $r_p= - 0.270199$
vCAM-1	NMR	urine	PSP-RS	negative	$p<0.67$, $r_p= - 0.151371$

vCAM-1	PLR	urine	PSP-RS	positive	$p<0.89$,<br>$r_p = 0.0483023$
vCAM-1	MHR	urine	PSP-RS	negative	$p<0.66$,<br>$r_p = -0.157742$
Tf	NHR	urine	CBS	positive	$p<0.54$,<br>$r_p = 0.253818$
Tf	NLR	urine	CBS	positive	$p<0.91$,<br>$r_p = 0.0458711$
Tf	NMR	urine	CBS	positive	$p<0.43$,<br>$r_p = 0.325647$
Tf	PLR	urine	CBS	negative	$p<0.35$,<br>$r_p = -0.380337$
Tf	MHR	urine	CBS	negative	$p<0.45$,<br>rp= -0.312649
**Tf**	**NHR**	**urine**	**PSP-P**	**positive**	**$p<0.04$,**<br>**$r_p = 0.632990$**
Tf	NLR	urine	PSP-P	positive	$p<0.73$,<br>$r_p = 0.125128$
Tf	NMR	urine	PSP-P	negative	$p<0.86$,<br>$r_p = -0.0604858$
Tf	PLR	urine	PSP-P	negative	$p<0.26$,<br>$r_p = -0.393677$
**Tf**	**MHR**	**urine**	**PSP-P**	**positive**	**$p<0.003$,**<br>**$r_p = 0.825357$**
Tf	NHR	urine	PSP-RS	negative	$p<0.51$,<br>$r_p = -0.235268$
Tf	NLR	urine	PSP-RS	negative	$p<0.78$,<br>$r_p = -0.100798$
Tf	NMR	urine	PSP-RS	negative	$p<0.48$,<br>$r_p = -0.252604$

Tf	PLR	urine	PSP-RS	positive	$p<0.70$, $r_p= 0.1352292$
Tf	MHR	urine	PSP-RS	negative	$p<0.67$, $r_p= - 0.1508558$

Legend: CBS – corticobasal syndrome; MHR - monocyte-to-high-density lipoprotein-cholesterol ratio; NHR - neutrophil-to-high-density lipoprotein ratio; NLR - neutrophil-to-lymphocyte ratio; NMR- neutrophile to monocyte ratio; PLR- platelet to lymphocyte ratio; PSP-P – progressive supranuclear palsy - parkinsonism predominant; PSP-RS- progressive supranuclear palsy- richardson's syndrome;Tf – transferrin; vCAM-1 - vascular cell adhesion molecule 1.
