# Supplementary material for: Potential Role of Transferrin and Vascular Cell Adhesion Molecule 1 in Differential Diagnosis Among Patients with Tauopathic Atypical Parkinsonian Syndromes
Source: Diagnostics (Basel). 2025 Oct 23;15(21):2676. doi: 10.3390/diagnostics15212676 (PMC12609891; doi:10.3390/diagnostics15212676)
Supplement: Supplementary file 1 [file diagnostics-15-02676-s001.zip › table S1 supplementary.pdf]

Table S1. Associations between serum vCAM-1 or transferrin levels and peripheral inflammatory ratios

SUBSTANCE	RATIO	BIOLOGICAL FLUID	APS SUBTYPE	TYPE OF CORRELATION	STATISTICAL PARAMETERS
**vCAM-1**	**NLR**	**serum**	**CBS**	**negative**	**$p<0.03$, r_p= - 0.739944**
vCAM-1	NHR	serum	CBS	negative	$p<0.07$, r_p= -0.657945
vCAM-1	NMR	serum	CBS	negative	$p<0.67$, r_p= -0.179605
vCAM-1	PLR	serum	CBS	negative	$p<0.77$ r_p= -0.122621
vCAM-1	MHR	serum	CBS	negative	$p<0.51$ r_p=-0.269648
vCAM-1	NLR	serum	PSP-P	positive	$p<0.25$ r_p= 0.251801
vCAM-1	NHR	serum	PSP-P	negative	$p<0.44$, r_p= -0.0569316
vCAM-1	NMR	serum	PSP-P	negative	$p<0.42$, r_p= -0.0719339
vCAM-1	PLR	serum	PSP-P	positive	$p<0.35$ r_p= 0.146730
vCAM-1	MHR	serum	PSP-P	positive	$p<0.45$ r_p= 0.041784
vCAM-1	NLR	serum	PSP-RS	negative	$p<0.21$ r_p= -0.386903
vCAM-1	NHR	serum	PSP-RS	positive	$p<0.66$, r_p= 0.141259
vCAM-1	NMR	serum	PSP-RS	positive	$p<0.85$, r_p= 0.0584292
vCAM-1	PLR	serum	PSP-RS	negative	$p<0.26$ r_p= -0.350515
vCAM-1	MHR	serum	PSP-RS	positive	$p<0.64$ r_p= 0.149823
**Tf**	**NHR**	**serum**	**CBS**	**negative**	**$p<0.04$, r_p= - 0.6445340**
Tf	NLR	serum	CBS	negative	$p<0.17$, r_p= - 0.377994
Tf	NMR	serum	CBS	positive	$p<0.33$, r_p= 0.181616
Tf	PLR	serum	CBS	positive	$p<0.17$, r_p= 0.389709
Tf	MHR	serum	CBS	negative	$p<0.10$, rp= -0.494329
Tf	NHR	serum	PSP-P	negative	$p<0.72$, r_p= -0.129827
Tf	NLR	serum	PSP-P	positive	$p<0.73$, r_p= 0.121513

Tf	NMR	serum	PSP-P	negative	$p<0.75$, $r_p = -0.115509$
Tf	PLR	serum	PSP-P	positive	$p<0.42$, $r_p = 0.283707$
Tf	MHR	serum	PSP-P	positive	$p<0.99$, $r_p = 0.003485$
Tf	NHR	serum	PSP-RS	positive	$p<0.87$, $r_p = 0.0495936$
Tf	NLR	serum	PSP-RS	positive	$p<0.68$, $r_p = 0.129386$
Tf	NMR	serum	PSP-RS	positive	$p<0.29$, $r_p = 0.330994$
Tf	PLR	serum	PSP-RS	positive	$p<0.96$, $r_p = 0.0146249$
Tf	MHR	serum	PSP-RS	positive	$p<0.94$, $r_p = 0.0236081$

Legend: CBS – corticobasal syndrome; MHR - monocyte-to-high-density lipoprotein-cholesterol ratio; NHR - neutrophil-to-high-density lipoprotein ratio; NLR - neutrophil-to-lymphocyte ratio; NMR- neutrophile to monocyte ratio; PLR- platelet to lymphocyte ratio; PSP-P – progressive supranuclear palsy - parkinsonism predominant; PSP-RS- progressive supranuclear palsy- richardson's syndrome;Tf – transferrin; vCAM-1 - vascular cell adhesion molecule 1.
