# Supplementary figures and images for: Potential Role of Transferrin and Vascular Cell Adhesion Molecule 1 in Differential Diagnosis Among Patients with Tauopathic Atypical Parkinsonian Syndromes
Source: Diagnostics (Basel). 2025 Oct 23;15(21):2676. doi: 10.3390/diagnostics15212676 (PMC12609891; doi:10.3390/diagnostics15212676)

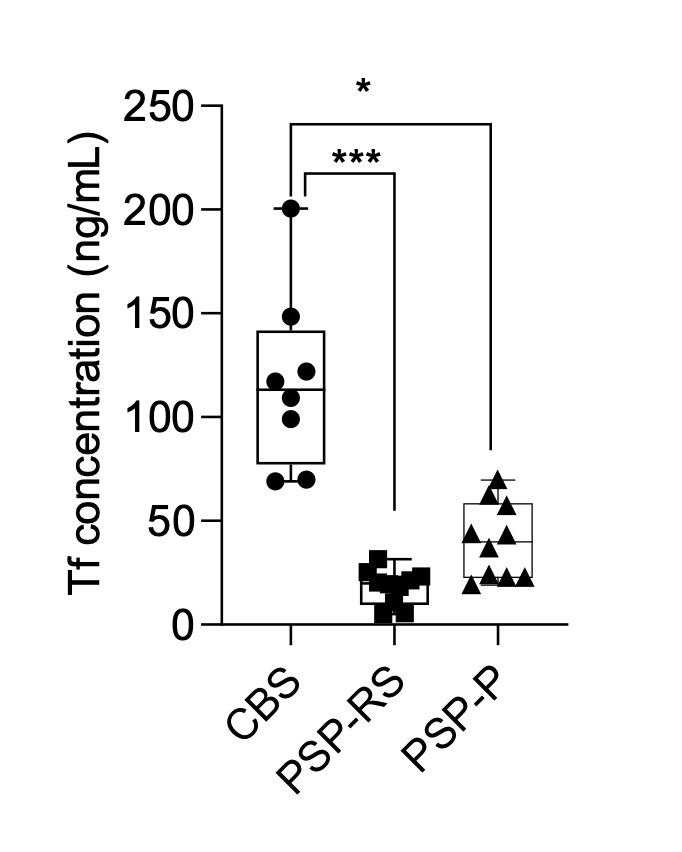

Supplement: Supplementary file 1 [file diagnostics-15-02676-s001.zip › Figure supplementary S1.JPG]

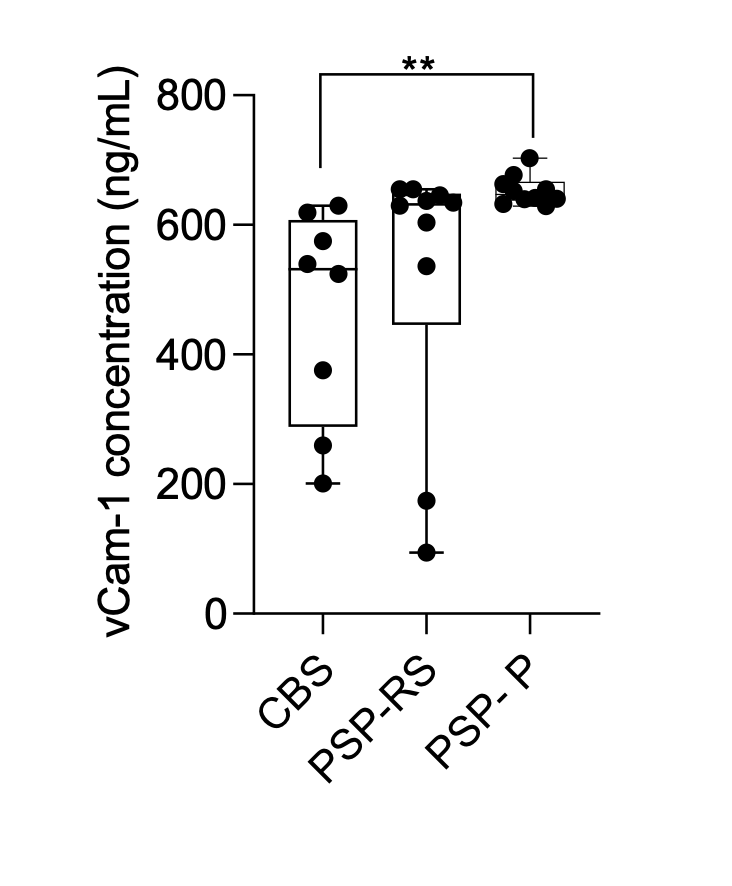

Supplement: Supplementary file 1 [file diagnostics-15-02676-s001.zip › Figure supplementary S2.JPG]

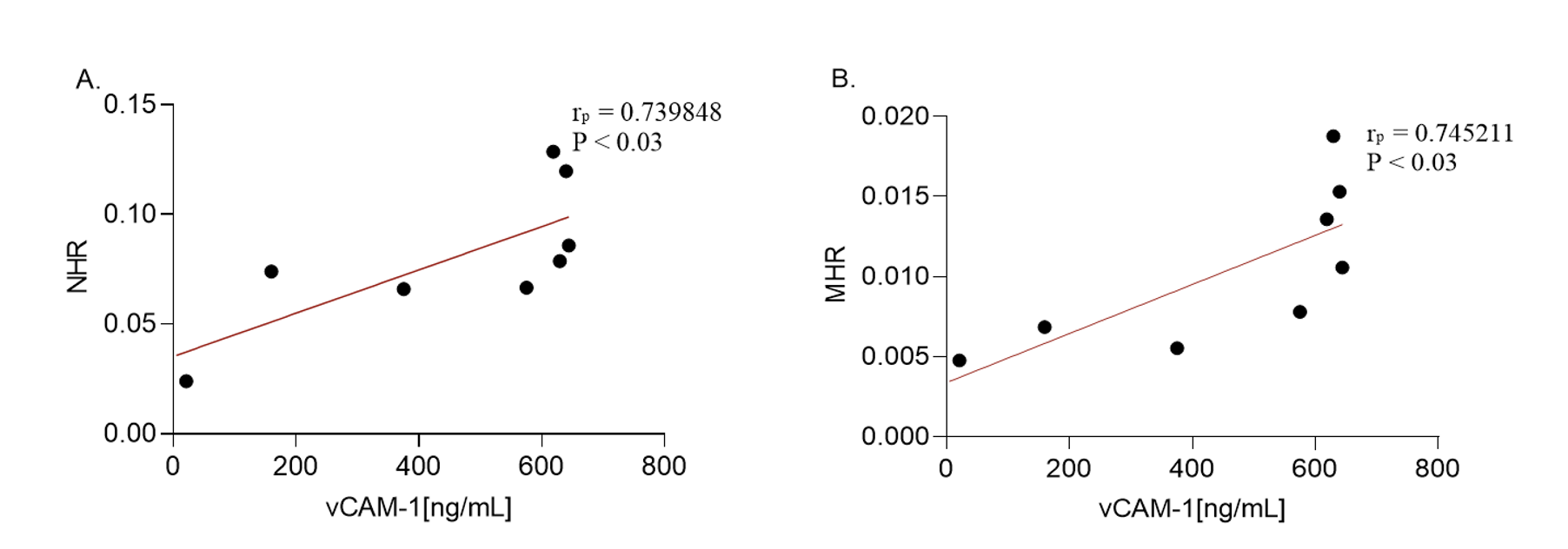

Supplement: Supplementary file 1 [file diagnostics-15-02676-s001.zip › Figure supplementary S3.JPG]

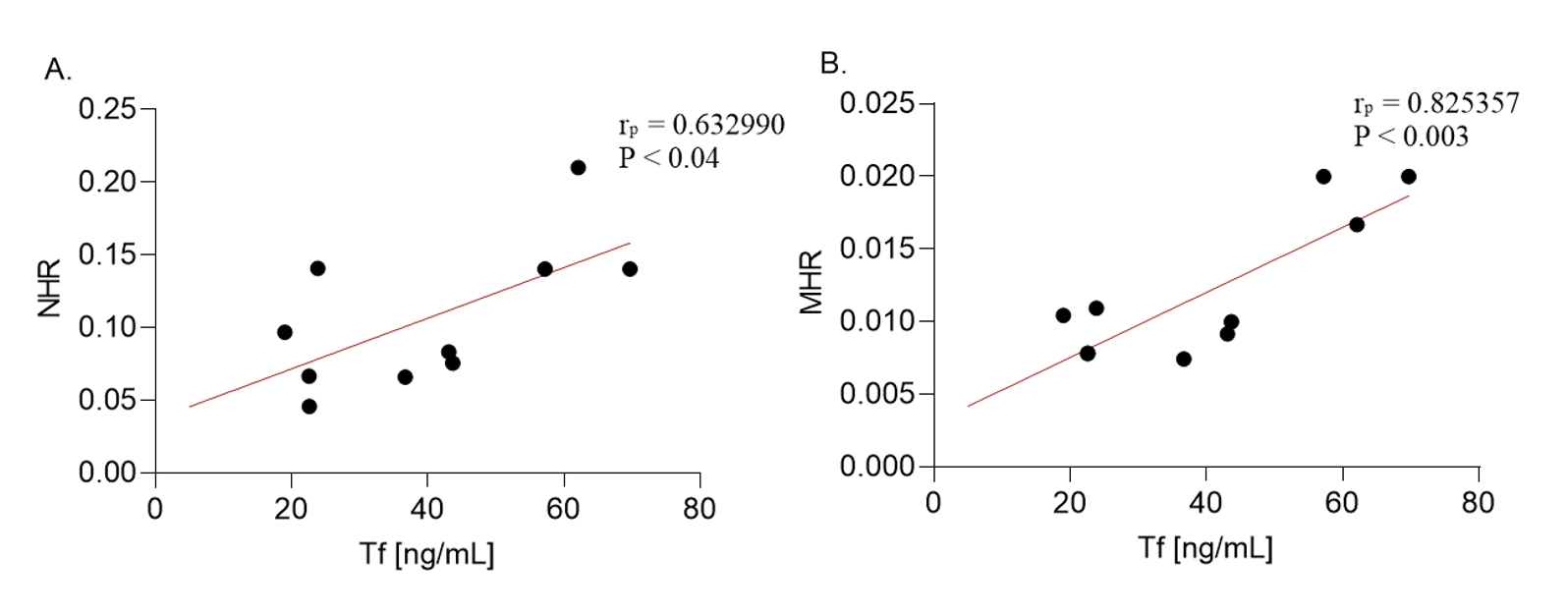

Supplement: Supplementary file 1 [file diagnostics-15-02676-s001.zip › Figure supplementary S4.JPG]
